# Determination of the embryotoxic effects of propofol injected into eggs on the cerebellum and spinal cord using histologic methods: an animal study

**DOI:** 10.55730/1300-0144.5760

**Published:** 2023-11-29

**Authors:** Murat İZGİ, Emrah SUR

**Affiliations:** 1 Department of Anaesthesiology and Reanimation, Faculty of Medicine, Hacettepe University, Ankara, Turkiye; 2 Department of Histology and Embryology, Faculty of Veterinary Medicine, Selçuk University, Konya, Turkiye

**Keywords:** Central nervous system, CHEST, chicken embryo, embryotoxicity, propofol

## Abstract

**Background/aim:**

This study aims to determine the possible embryotoxic effects of propofol on the cerebellum and spinal cord using fertile chicken eggs.

**Materials and methods:**

A total of 430 fertile eggs were divided into 5 groups: control, saline, 2.5 mg.kg^−1^, 12.5 mg.kg^−1^, and 37.5 mg.kg^−1^ propofol. Injections were made immediately before incubation via the air chamber. On the 15th, 18th, and 21st day of incubation, 6 embryos from each group were evaluated. Serial paraffin sections taken from the cerebellum and spinal cord were stained with hematoxylin–eosin, Kluver–Barrera, toluidine blue, and periodic acid-Schiff’s reaction. The outer granular layer and total cortex thickness were measured, and the linear density of the Purkinje cells was determined. The ratios of the substantia grisea surface area to the total surface area of the spinal cord were calculated. The transverse and longitudinal diameters of the canalis centralis were also assessed.

**Results:**

No structural malformation was observed in any embryos examined macroscopically. No significant difference was observed between the groups in terms of development and histologic organization of the cerebellum and spinal cord. However, on the 15th, 18th, and 21st day, the outer granular layer (p < 0.001 for all days) and the total cortex thickness (p < 0.01, p < 0.001, and p < 0.001, respectively) decreased significantly in different propofol dose groups in varying degrees in the cerebellum. Similarly, in the spinal cord, there were significant changes in the ratios of the substantia grisea surface area to the total surface area (p < 0.01 and p < 0.001, respectively).

**Conclusion:**

It was concluded that the in-ovo-administered propofol given immediately before incubation has adverse effects on the developing cerebellum and spinal cord. Therefore, it is important for anesthesiologists always to remain vigilant when treating female patients of childbearing age.

## 1. Introduction

Exposure to anesthetic agents may occur during general anesthesia or the sedation required for surgical or interventional procedures – and for sedation in intensive care units (ICUs). During the sedation process in ICUs, the duration and, therefore, the amount of anesthetic agent exposure is many times greater than the duration of the surgical procedure [1].

Propofol is among the most commonly used intravenous anesthetics in clinical anesthesia and ICUs [1]. Propofol, which has hypnotic, sedative, and antiemetic properties, was first used clinically by Kay and Rolly in 1977 [2]. It is a fat-soluble and low-molecular-weight non-ionizing agent, has rapid transplacental passage, and can cross the blood-brain barrier. Propofol is cleared very quickly from the circulation of the mother and newborn [3]. Recent studies have shown that it may cause neurotoxicity in the developing brain [4–9]. Mechanisms such as calcium signaling, mitochondrial fusion, neuroinflammation, microRNAs, and neurotrophin expression deregulation have been shown to underlie propofol-induced detrimental effects. Propofol exposure has been reported to significantly induce neuroapoptosis and negatively affect neural stem cell proliferation, neurogenesis, dendrite development, and cognitive development in developing animal and human stem cell models [10]. In another study, the authors determined that propofol activated cell apoptosis in neural stem cells in a dose-dependent manner through miR-9-5p/CXCR4 signaling [1]. Pearn et al.’s study found that propofol caused growth-cone collapse, axonal transport disorder, loss of synaptic connection, and behavioral deficiencies in newborn mice [11].

The frequency of nonobstetric surgery performed during pregnancy varies between 0.3% and 2.2%, corresponding to ~100,000 cases per year in the United States and European Union countries [12]. Considering the points needing consideration if the pregnancy status of these patients is known, a period exists when the pregnancy is not known, even by the expectant mother, which is between the 15th and 56th days (2–8 weeks) of gestation, corresponding to the embryogenesis period when the embryo is most sensitive to embryotoxic and teratogenic substances. Due to the teratogenic effects of the exposed agents, embryos may be lost, or various structural and developmental anomalies may be observed during this period. Although it is generally accepted that propofol is not a teratogenic agent at clinical doses, it is impossible to conduct human teratogenicity studies due to ethical concerns [13].

The use of fertile chicken eggs in embryotoxicity and teratogenicity tests was first described by Jelinek in 1977. The process has developed over time and continues today [14]. According to the study’s aims, substance injection can be made at different periods of incubation and in other parts of the egg. Injections can be made to the yolk sac, subgerminal space, allantois, amnion, or air chamber, depending on the physicochemical properties of the active substance and solvent tested – and according to the researcher’s preference. The first 72 h of chick embryogenesis are essential for subsequent development success. During this period, organ systems develop rudimentarily in an appropriate relationship with others. Therefore, to observe the most pronounced effects of teratogens, the active substance in question should be administered to the embryos in the first 72 h.

In studies using the air chamber injection method, the prepared solution was used in a volume of 50 or 100 μL [15,16]. It is recommended that the tested substance be used in at least 3 different doses [17]. The chicken embryo developmental stages scale, introduced by Viktor Hamburger and Howard L. Hamilton, is used to determine the developmental stages of the embryos [18].

This study aims to determine the possible effects of propofol, a frequently preferred general anesthetic agent in clinical practice, on the embryonic development of the central nervous system (CNS) using fertile chicken eggs. The study’s primary purpose is to determine the possible embryotoxic effects of propofol on the cerebellum and spinal cord at the light microscopic level. Additional objectives are evaluating the macroscopic defects on the CNS, such as neural tube closure defects, if present, and determining if the mortality rate changes with increasing doses of propofol.

## 2. Materials and methods

### 2.1. Ethical approval

Ethical approval was provided by the Ethical Committee of Selçuk University Veterinary Faculty’s Experimental Animal Production and Research Center, Konya, Türkiye, on 29 March 2016 with certificate number 2016/40.

### 2.2. Propofol test solution

An intravenous (IV) injectable propofol solution (Propofol 2%, Fresenius, Germany) was used to prepare the test solutions.

### 2.3. Egg material

In the study, 430 fertile eggs from Atabey breed chickens were used. Eggs with an average weight of 50–55 g were preferred for the study [19–21].

### 2.4. Treatment groups, preparation of the test solution, and embryonic exposure

The selected eggs were divided into 5 groups:

**Control group** (G1, 55 eggs): No injection was made to these eggs.
**Solvent–control group** (G2, 60 eggs): These eggs were injected with 0.9% NaCl at 100-μL volume via the air chamber.
**2.5 mg.kg^−1^ propofol-injected group** (G3, 90 eggs): These were injected with 2% propofol at a dose of 2.5 mg.kg^−1^ (6.25 μL 2% Propofol + 93.75 μL 0.9% NaCl) at 100-μL volume via the air chamber [19].
**12.5 mg.kg^−1^ propofol-injected group** (G4, 100 eggs): These were injected with 2% propofol at a dose of 12.5 mg.kg^−1^ (31.25 μL 2% propofol + 68.75 μL 0.9% NaCl) at 100-μL volume via the air chamber [19].
**37.5 mg.kg^−1^ propofol-injected group** (G5, 125 eggs): These were injected with 2% propofol at a dose of 37.5 mg.kg^−1^ (93.75 μL 2% propofol + 6.25 μL 0.9% NaCl) at 100-μL volume via the air chamber [19].

The blunt ends of the eggs, except for those in the control group, were disinfected with 96% ethanol before injection and drilled with an egg drill. All injection procedures were performed under sterile conditions in a laminar flow cabinet with a sterile-tipped micropipette at 100 μL/egg volume and through the eggs’ air chamber. The holes were sealed with melted paraffin immediately after the injections. Subsequently, the eggs were settled in the incubator under optimal conditions (incubation temperature: 37.7 °C; relative humidity: 65%; turning 180° once every 2 h) [19,21].

### 2.5. Collection of tissue samples and histological investigations

On the 15th and 18th day of incubation, 6 live embryos obtained from randomly selected eggs from each group were weighed. Their developmental stages were determined according to the Hamburger–Hamilton (HH) scale [18]. The embryos and chickens were sacrificed by decapitation [22], and cerebellum and spinal cord tissue samples were taken. Tissue samples taken from the cerebellum and spinal cord of 6 embryos and 6 chicks from each group hatched on the 21st day were fixed in a 10% buffered formol saline (pH 7.4) solution and analyzed [22]. The tissues were followed using routine histologic techniques and embedded in paraffin. Serial sections at a thickness of 6 μm were taken from the paraffin blocks [20–28]. The serial sections of 6-μm thickness were taken at a minimum of 30-μm intervals from the cerebellum, and the serial sections at a minimum of 100-μm intervals from the spinal cord. The sections were stained with hematoxylin–eosin, Kluver–Barrera, and toluidine blue [29]. In addition to these methods, periodic acid-Schiff’s reaction was applied to examine the cells forming the corpus gelatinosum in the lumbar segment of the spinal cord [29]. The slides were examined under a Leica DM-2500 (Leica Microsystems, Wetzlar, Germany) model light microscope using a DFC-320 camera attachment, and digital images of the regions required for histometric measurements were recorded.

### 2.6. Histometric analyses

#### 2.6.1. Cerebellum

**Figure 1:**
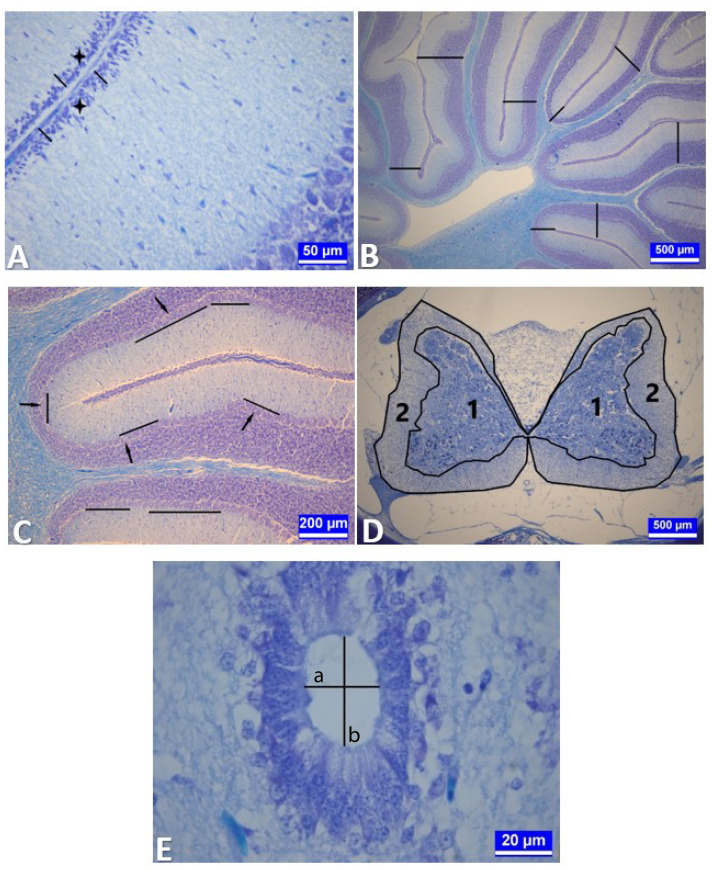
Histometric measurements of cerebellum and spinal cord tissues. **A:** Measurement of the thickness of the outer granular layer in a section of cerebellum tissue. A: Stars: Outer granular layer. Black bars: Measurement points. Kluver–Barrera staining. Bar: 50 μm. **B:** Measurement of total cortex thickness in a section of cerebellum tissue. Black bars: Measurement points. Kluver–Barrera staining. Bar: 500 μm. **C:** Counting Purkinje cells in a section of cerebellum tissue. Arrows: Purkinje cells. Black bars: Counted regions. Kluver–Barrera staining. Bar: 200 μm. **D:** A section through the lumbar segment of the medulla spinalis. Area measurements were made in the regions bounded by black lines 1: Substantia grisea. 2: Substantia alba. Kluver–Barrera staining. Bar: 500 μm. **E:** Measurements of the transverse (black bar a) and longitudinal (black bar b) diameters of the canaliculus centralis in a section from the thoracic segment of the medulla spinalis. Bar: 20 μm.

While the outer granular layer (Figure 1A) and total cortex thickness were measured from at least 20 different regions in 3 serial sections taken from the midline of each chick’s cerebellum tissue (Figure 1B), the linear density of the Purkinje cells was also determined at 1-mm length in different regions of each serial section [27,30] (Figure 1C).

#### 2.6.2. Spinal cord

The spinal cord sections were evaluated in 3 different segments: cervical, thoracic, and lumbar. The ratios of the substantia grisea surface area to the total surface area of the spinal cord were calculated in 3 serial sections in different segments of each chick [21] (Figure 1D) (the canalis centralis was included in the surface areas in the measurements).

The transverse and longitudinal diameters of the canalis centralis were evaluated in serial sections of the spinal cord tissue taken from 3 different segments [21] (Figure 1E). All histometric measurements were performed using the Leica IM50 measurement program (Leica Microsystems, Wetzlar, Germany).

### 2.7. Statistical analyses

SPSS 15.0 (Statistical Package for the Social Sciences, IBM, New York, USA) was used to statistically evaluate all data. Variance analysis was used to assess outer granular layer thickness, total cortex thickness, the substantia grisea surface area ratios to the total surface area, and transverse and longitudinal diameters of the canalis centralis. Differences between groups were determined using Tukey’s test. The chi-square test was used to determine the significance of the difference between groups in terms of Purkinje cell count.

## 3. Results

The rates of dead embryos were as follows: 16% for G1, 28.8% for G2, 46.4% for G3, 31.2% for G4, and 73.2% for G5. The HH stages of dead embryos between G5 and G3 (p < 0.001) and G5 and G1 (p = 0.041) for day 21 were statistically significant. Embryonic deaths concentrated at HH 20 (21.2%) and 40 (20.4%) during the incubation period. No structural anomaly/malformation was observed in any embryos examined macroscopically.

### 3.1. Histological and histometric findings

#### 3.1.1. Fifteenth day of incubation

##### 3.1.1.1. Cerebellum

**Figure 2:**
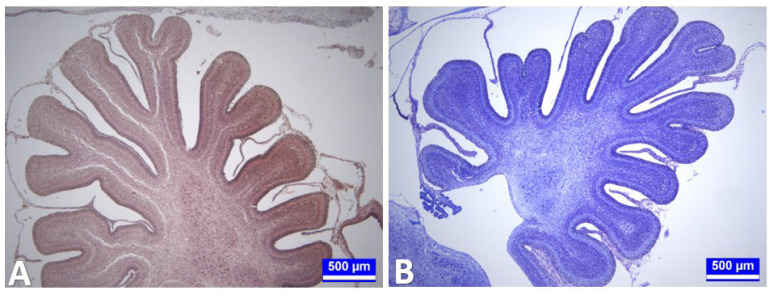
Cerebellum tissue sections on the 15th day of incubation. A: Cerebellum tissue section of a control (G1) embryo on day 15 of incubation. General view of the developing cerebellum. Triple staining. Bar: 500 μm. B: Cerebellum tissue section from an embryo obtained from an egg injected with 37.5 mg.kg^−1^ propofol (G5) on day 15 of incubation. Kluver–Barrera staining. Bar: 500 μm.

On the 15th day of incubation, the folia of the cerebellum were clearly distinguished. The cortex layer consisted of a very thick outer granular layer, a primitive molecular layer, an inner cortical layer, and an inner granular layer. The Purkinje cells formed the inner cortical layer with an almost single-row arrangement. Although no detectable difference was observed between the experimental and control groups in terms of histologic organization (Figure 2), histometric measurements revealed that outer granular layer thickness decreased significantly in all experimental groups, compared to G1 and G2 (p < 0.001). Total cortex thickness was significantly reduced only in G5 compared to all other groups (p < 0.01, Table 1). There was no statistically significant difference between the experimental groups compared to G1 and G2 in terms of Purkinje cell count (p > 0.05).

##### 3.1.1.2. Spinal cord

During this period, the substantia grisea region and the surrounding substantia alba layer, with its classic “butterfly” appearance, were in the middle in sections taken from the cervical, thoracic, and lumbar spinal cord segments in all groups. The motor cells in the ventral corn could be distinguished from other cells. It was observed that the ependymal cells were lined in shapes ranging from cubic to prismatic inside the round-oval-shaped canalis centralis. The sinus rhomboideus, which distinguishes the lumbar segment from other segments and separates the right and left dorsal corns in the dorsal of the spinal cord in birds, and the corpus gelatinosum, located in this sinus, contain a small number of glial cells containing glycogen granules.

**Figure 3:**
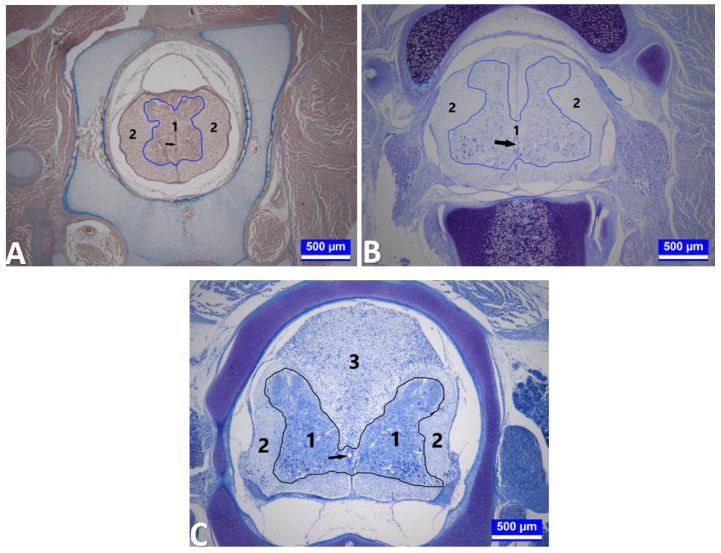
Medulla spinalis tissue sections on the 15th day of incubation. **A:** A section through the cervical segment of the medulla spinalis of an embryo from the group injected with 12.5 mg.kg–1 propofol (G4) on day 15 of incubation. 1 (area bounded by blue lines): Substantia grisea. 2: Substantia alba. Arrow: Canalis centralis. Triple staining. Bar: 500 μm. **B:** A section through the thoracic segment of the medulla spinalis of an embryo from G2 on day 15 of incubation. 1 (area bounded by blue lines): Substantia grisea. 2: Substantia alba. Arrow: Canalis centralis. Kluver–Barrera staining. Bar: 500 μm. **C:** A section through the lumbar segment of the medulla spinalis of an embryo from the group injected with 12.5 mg.kg^−1^ propofol (G4) on day 15 of incubation. 1 (area bounded by black lines): Substantia grisea. 2: Substantia alba. 3: Corpus gelatinosum. Arrow: Canalis centralis. Toluidine blue. Bar: 500 μm.

In histometric measurements, it was determined that the ratio of the surface area of the substantia grisea to the total surface area of the spinal cord observed in the sections increased in the cervical segments of the embryos in the G4 (p < 0.001) and decreased in the thoracic segments of the embryos in the G4 (p < 0.001), compared to G1 and G2. However, G3 and G5 did not significantly change, compared to G1 and G2, in both segments (Table 2, Figure 3).

**Figure 4:**
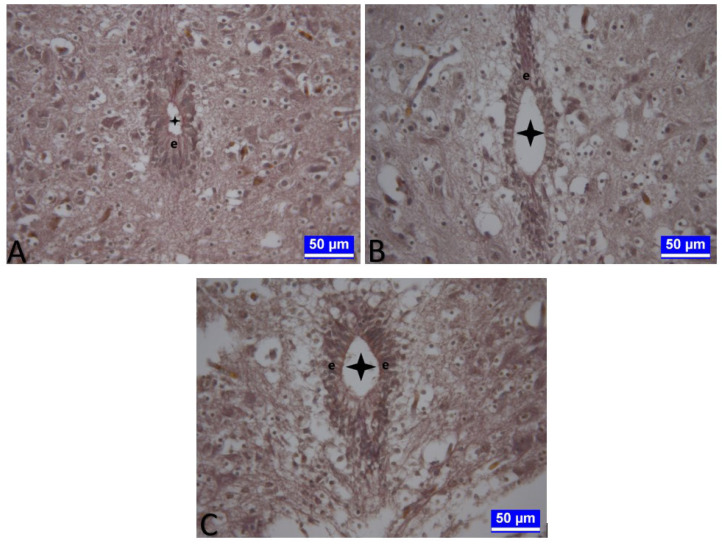
Medulla spinalis tissue sections on day 15 of incubation. **A:** A section through the cervical segment of the medulla spinalis of a control embryo (G1) on day 15 of incubation. A: Asterisk: Canalis centralis. e: Ependymal cells. Triple staining. Bar: 50 μm. **B:** A section through the thoracic segment of the medulla spinalis of an embryo from the control group (G1) on day 15 of incubation. A: Asterisk: Canalis centralis. e: Ependymal cells. Triple staining. Bar: 50 μm. **C:** A section through the lumbar segment of the medulla spinalis of an embryo from G2 on day 15 of incubation. Asterisk: Canalis centralis. e: Ependymal cells. Triple staining. Bar: 50 μm.

The longitudinal diameters of the canalis centralis in the embryos belonging to G4 decreased in the thoracic segments (p < 0.01, Figure 4), whereas the transverse diameter increased in the lumbar segments of the embryos in the same group, compared to G1 (p < 0.05, Table 3). During this period, it was observed that the vertical diameters of the embryos obtained from the eggs in G2 increased in the lumbar segments compared to G1 (p < 0.001, Table 3).

#### 3.1.2. Eighteenth day of incubation

##### 3.1.2.1. Cerebellum

**Figure 5:**
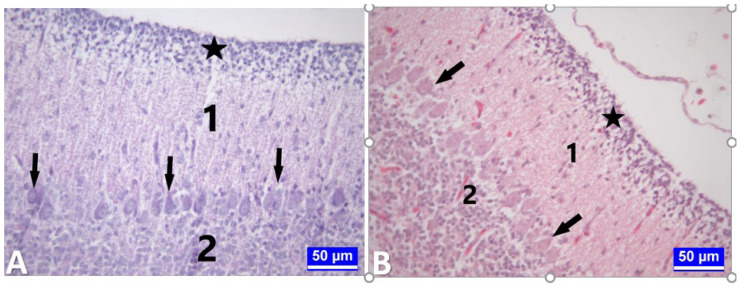
Cerebellum tissue sections on day 18 of incubation. **A:** Cerebellum tissue section of a control embryo (G1) on day 18 of incubation. A: Star: Outer granular layer. 1: Stratum moleculare. 2: Stratum granulosum. Arrows: Purkinje cells (stratum gangliosum). periodic acid-Schiff’s reaction (PAS) reaction. Bar: 50 μm. **B:** Cerebellum tissue section from an embryo obtained from an egg injected with 12.5 mg.kg^−1^ propofol (G4) on day 18 of incubation. Asterisk: Outer granular layer. 1: Stratum moleculare. 2: Stratum granulosum. Arrows: Purkinje cells (stratum gangliosum). Hematoxylin–Eosin. Bar: 50 μm.

On the 18th day of incubation, it was observed that the histologic organization of the cerebellum was almost complete. It was also noted that the outer granular layer became thinner compared to the previous period. Although no significant difference was observed between the experimental and control groups in terms of histologic organization, as a result of the histometric measurements, it was determined that the outer granular layer and total cortex thicknesses decreased significantly in all experimental groups, compared to G1 (p < 0.001, Table 1, Figure 5). Additionally, during this period, the thickness of the outer granular layer decreased in G2 compared to G1 (p < 0.001). There was no statistically significant difference between the experimental groups, compared to G1 and G2, in terms of Purkinje cell count (p > 0.05).

##### 3.1.2.2. Spinal cord

During this period, it was observed that the development of the spinal cord continued in all groups, and both substantia grisea and substantia alba were enlarged, compared to the previous period. The presence of the corpus gelatinosum in the lumbal segment was also more evident.

**Figure 6:**
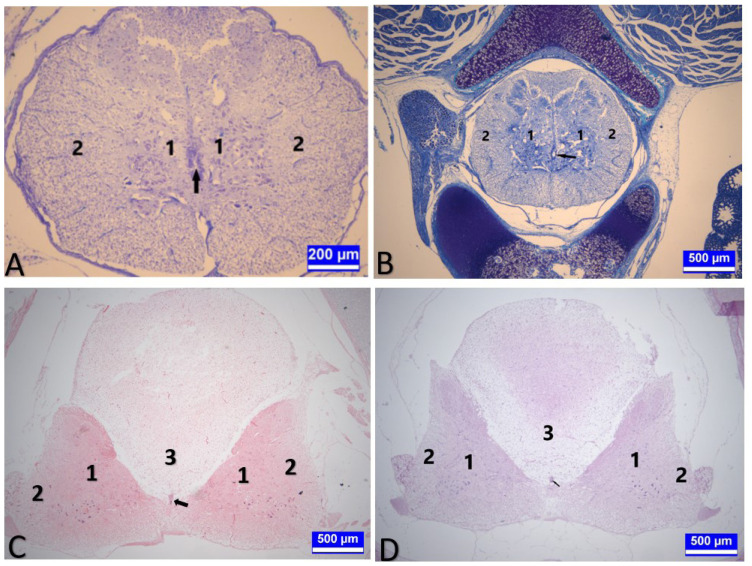
Medulla spinalis tissue sections on the 18th day of incubation. **A:** A section of the cervical segment of the medulla spinalis of an embryo obtained from the group treated with 2.5 mg.kg^−1^ propofol (G3) on day 18 of incubation. 1: Substantia grisea. 2: Substantia alba. Arrow: Canalis centralis. Kluver–Barrera staining. Bar: 200 μm. **B:** A section through the thoracic segment of the medulla spinalis of an embryo from the group treated with 37.5 mg.kg^−1^ propofol (G5) on day 18 of incubation. 1: Substantia grisea. 2: Substantia alba. Arrow: Canalis centralis. Toluidine blue. Bar: 500 μm. **C:** A section through the lumbar segment of the medulla spinalis of a control embryo (G1) on day 18 of incubation. 1: Substantia grisea. 2: Substantia alba. 3: Corpus gelatinosum. Arrow: Canalis centralis. PAS reaction. Bar: 500 μm. **D:** A section through the lumbar segment of the medulla spinalis of a chick from the group treated with 2.5 mg.kg^−1^ propofol (G3) on day 21 of incubation. 1: Substantia grisea. 2: Substantia alba. 3: Corpus gelatinosum. Arrow: Canalis centralis. PAS reaction. Bar: 500 μm.

As a result of the histometric measurements, it was determined that the ratio of the surface area of the substantia grisea to the total surface area of the spinal cord in the sections increased in the cervical segments of the embryos in G3 compared to G1; however, it decreased in the same segments of the embryos in G5 compared to G1 (p < 0.001, Table 2, Figure 6). It was noted that this ratio also decreased in the thoracic segments of the embryos in the G4 and G5, compared to G1 (p < 0.01). Similarly, this rate decreased in the cervical segments of the embryos obtained from the G2, compared to G1 (p < 0.001).

The transverse diameters of the canalis centralis in the embryos belonging to G4 were enlarged in the cervical segments (p < 0.01), and the transverse diameters of the embryos in the G3 were enlarged in the thoracic segments, compared to G1 (p < 0.01, Table 3). During this period, it was determined that the transverse diameter of the canalis centralis increased in the thoracic and lumbar segments of the embryos obtained from the G2, compared to G1 (p < 0.01 and p < 0.001, respectively).

#### 3.1.3. First day of hatching (incubation day 21)

##### 3.1.3.1. Cerebellum

**Figure 7:**
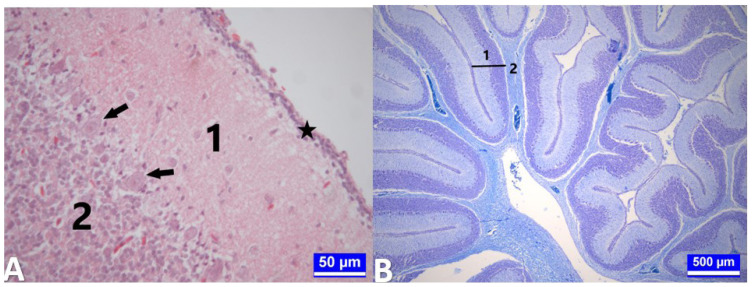
Cerebellum tissue sections on hatching day. **A:** Cerebellum tissue section from a chick injected with serum physiological (G2) on day 21 of incubation. Asterisk: Outer granular layer. 1: Stratum moleculare. 2: Stratum granulosum. Arrows: Purkinje cells (stratum gangliosum). Hematoxylin–Eosin. Bar: 50 μm. **B:** Cerebellum tissue section from a chick injected with 2.5 mg.kg^−1^ propofol (G3) on day 21 of incubation. 1 (Bar): Cortex. 2: Medulla. Kluver–Barrera staining. Bar: 500 μm.

In the cerebellum tissue sections obtained from the chicks from all groups on day 21 of incubation (the first day of hatching), the outer granular layer, which had thinned considerably, was still present. The classical cellular organization of the cerebellum was more distinct (Figure 7).

Histometric measurements revealed that the thickness of the outer granular layer and total cortex decreased significantly in all experimental groups compared to G1 (p < 0.001, Table 1). During this period, the total cortex thickness of the chicks in G2 also decreased significantly compared to G1 (p < 0.001). Similar to the previous periods, there was no statistically significant difference between the experimental groups, compared to G1 and G2, in terms of Purkinje cell numbers in this period (p > 0.05).

##### 3.1.3.2. Spinal cord

During this period, it was observed that the spinal cord had almost gained its classical histological organization in all groups, especially the corpus gelatinosum in the lumbar segment, which was enlarged, and the canalis centralis remained within this structure. The ratio of the surface area of the substantia grisea to the total surface area of the medulla spinalis was found to have increased, especially in the lumbar segments of the embryos in G3 and G5, compared to G1 (p < 0.01, Table 2, Figure 6). The transverse diameter of the canalis centralis was enlarged, especially in the cervical segments of the embryos in G4 and G5, compared to G1 (p < 0.01, Table 3).

## 4. Discussion

The most important reason why anesthetic agent exposure is important during pregnancy is that maternal practices may directly or indirectly affect the fetus. The situation causing the most concern for the fetus is the potential for teratogenicity. The highest risk of teratogenicity in humans is 15–56 days from the mother’s last menstrual period before conception, the organogenesis period when the fetus’s organs are formed [31]. This period also includes a stage in which the mother is unaware of her pregnancy. Miscarriage, fetal death, structural anomalies, growth retardation, or functional deficiencies may occur as a result of teratogenic effects. Although avoiding elective surgery in pregnant patients is recommended – and, if possible, also performing surgery in the second trimester of pregnancy, which is the optimal time interval [31] – there is little chance of postponing emergency surgical intervention. It has been reported that there is a significant relationship between first-trimester maternal general anesthesia exposure and the risk of CNS defects associated with other major defects [32].

Because the development process of the chicken embryo, especially in the first 48 h of early development, is similar to the development of the human embryo in the 1st month, fertile chicken eggs are a suitable model to examine the embryotoxicity and teratogenicity studies [27,33–45]. The embryonic development and histologic organization of the spinal cord and cerebellum are well known and provide advantages in terms of histometric measurements. Particularly in chickens, the CNS tissue constitutes a comparable model with mammals when histologic differentiation stages are considered [46].

Fertile chicken eggs are cheap, easy to access and manipulate, and provide an advantage over other laboratory animals in terms of statistical evaluations because they can be used in large numbers. They also comply with legal restrictions, ethical rules, and animal rights, minimizing the pain inflicted on the living organism. To demonstrate the validity of the Chicken Embryotoxicity Screening Test (CHEST), 50 different chemicals were tested in rabbits and rats, and the results were found to be 80% consistent with CHEST; however, although alternative test methods cannot replace routine test methods, they will only help and guide these tests [47].

In the present study, histometric measurements made in the cerebellum of embryos in all periods showed that the outer granular layer thickness and total cortex thickness decreased in a dose-dependent manner in all 3 dose groups. It was also noted that the thickness of the outer granular layer on the 18th day of incubation, and the total cortex thickness on the 21st day of incubation, decreased in G2 compared to G1. In addition to the fact that the main function of the cerebellum is the coordination of voluntary motor movements, motor learning, and balance, various studies have shown that it affects cognitive functions, visuospatial functions, language functions, emotional stability, and personality traits [48]. Lee et al. examined the effects of propofol on the synaptic responses of cerebellar Purkinje cells, showing that propofol exposure might adversely affect synaptic messages in the cerebellar cortex and complex brain functions [49]. In 2 studies examining the neurotoxic effects of propofol in a zebrafish model, it was reported that exposure to propofol increased apoptosis in oligodendrocytes, caused a decrease in myelin basic protein expression, and had neurotoxic effects [50,51]. A study examining fetal and neonatal primate brain tissue following propofol exposure reported that propofol exposure significantly increased apoptosis in oligodendrocytes and neurons [6]. In a study conducted on mice, it was observed that the administration of propofol to pregnant mice caused retardation in the development of physical and neurologic reflexes in the offspring [52]. In a recent study by Bleeser et al., in which the authors evaluated 65 preclinical studies, they reported that anesthetic agent exposure during pregnancy impaired learning and memory and caused neuronal damage in all experimental models and that apoptosis was the main mechanism in the effects of propofol [53]. In another study conducted in pregnant rats, it was suggested that exposure to propofol during pregnancy impaired neuronal development, which might be associated with depression and anxiety-like behaviors and cognitive disorders in offspring [54]. In a study evaluating the neurocognitive effects of fetal anesthetic exposure, it was reported that increased neuroapoptotic activity due to exposure to GABA receptor agonists (such as propofol) and NMDA receptor antagonists might have long-term and harmful consequences in developing fetuses [55]. In a study by Landau et al., it was reported that increased expression of behavioral problems was encountered in children exposed to general anesthetics during pregnancy [56]. In this study, the decreases detected in the total cortex thickness and outer granular layer thickness of the cerebellum tissue may have caused adverse effects on cerebellar functions.

The spinal cord forms the part of the CNS located in the columna vertebralis. The spinal cord consists of the inner substantia grisea and outer substantia alba. There are motor neurons in the anterior horn of the substantia grisea and sensory neurons in the posterior horn [28,57,58]. Neural tube defects, especially malformations, including closure defects of the spinal cord, can be seen as the teratogenic effects of several drugs used today. The effects of papaverine [33], diazepam [34,36,59], local anesthetics [37,39], caffeine [35], pregabalin [45], rizatriptan [44], Botox [60], sodium phenytoin [41], lacosamide [61], levetiracetam [62], meloxicam [42], methotrexate [40], and verapamil [38] on the neural tube have been investigated by various researchers using fertile chicken eggs. As a result of these studies, it was observed that most agents, except for Botox and levetiracetam, cause neural tube defects. In the present study, although no malformation was observed in the spinal cord, it was observed that there were differences at various levels in the ratio of the substantia grisea to the total medulla spinalis surface area in the experimental groups. As a result of the changes observed in the substantia grisea, it was possible to notice defects in the motor and sensory functions of the spinal cord. In addition, while there were differences in central canal diameters in the propofol-injected groups, the fact that the central canal diameters were affected in G2 suggests that the embryo was extremely sensitive in the early stages of development and that even the use of saline might have some effects similar to the use of a drug. Changes in central canal diameters will affect the circulation of cerebrospinal fluid (CSF) [10]. The main function of CSF is to provide mechanical and immunologic protection to the brain and spinal cord. Considering that the factors affecting the free circulation of CSF may cause serious dysfunctions in the later stages of life, the significance of the findings obtained from this study will increase.

The study had some limitations. Clearer data could be obtained using Caspase-3 and TUNEL to detect apoptosis in the relevant tissues among the staining methods. In addition, observing the chicks for longer periods after they have hatched will make it possible to evaluate whether their cognitive functions are compatible with their development. Thus, the clinical reflections of the data we obtained could be better observed in the short and long term.

It was observed that propofol injected into fertile chicken eggs immediately before incubation caused no macroscopic malformation on the CNS, but it caused significant differences in the cerebellum and spinal cord due to histometric measurements made at the light microscopic level. To our knowledge, no other study exists that evaluates propofol’s embryotoxic effects on the cerebellum and spinal cord using histologic methods.

Propofol, an IV agent used in general anesthesia and sedation, is used extensively in Türkiye and worldwide for patients of all age groups. Especially in female patients of childbearing age, the pregnancy status of the patient should be questioned, and if pregnancy is diagnosed in the first trimester and the surgery is elective, postponing the surgery to the second trimester should be strongly considered. In addition, there is the possibility of performing surgery at a time when the mother is not even aware of her pregnancy. This period is the most important in organogenesis and the most sensitive to external factors. Therefore, it is essential for anesthesiologists always to be aware of female patients of childbearing age, request pre-operative ß-HCG tests if necessary, or, if this is impossible, accept the patient as pregnant and adjust their drug-dose preferences accordingly.

## Figures and Tables

**Table 1:** Findings obtained from histometric measurements on the cerebellum tissues on the 15th, 18th, and 21st day of incubation.

**15th day**	**GROUP**	**OGL**	**TC**	**PCC**
	**G1**	64.32 ± 1.10^a^	186.91 ± 2.89^a^	53.45 ± 1.36
	**G2**	61.98 ± 0.98^a^	186.19 ± 3.04^a^	51.25 ± 1.10
	**G3**	**55.41 ± 0.95** b	184.29 ± 2.88^ab^	48.23 ± 1.55
	**G4**	**53.38 ± 1.24** bc	176.73 ± 2.91^ab^	49.05 ± 1.57
	**G5**	**50.53 ± 1.12** c	**174.29 ± 3.39** b	48.87 ± 1.09
		***	**	NS
**18th day**	**G1**	53.83 ± 1.30^a^	322.84 ± 4.38^a^	43.32 ± 1.36
	**G2**	**42.10 ± 0.82** b	310.57 ± 4.33^a^	44.33 ± 1.75
	**G3**	**36.74 ± 0.89** c	**276.55 ± 3.50** b	42.79 ± 1.23
	**G4**	**37.66 ± 0.95** c	**250.00 ± 3.88** c	38.90 ± 1.21
	**G5**	**34.32 ± 0.99** c	**249.49 ± 3.32** c	38.20 ± 0.83
		***	***	NS
**21st day**	**G1**	21.52 ± 0.38^a^	362.03 ± 4.37^a^	37.32 ± 0.81
	**G2**	21.13 ± 0.33^a^	**337.72 ± 4.74** b	38.14 ± 0.74
	**G3**	**18.16 ± 0.41** b	**336.34 ± 4.56** b	34.96 ± 0.73
	**G4**	**17.85 ± 0.36** b	**310.22 ± 4.89** c	37.67 ± 0.80
	**G5**	**16.90 ± 0.36** b	**310.66 ± 4.38** c	34.61 ± 0.69
		***	***	NS

NS: Not significant;

* p < 0.05;

** p < 0.01;

*** p < 0.001;

a, b Different letters in the same column are statistically significant;

G1: Control; G2: Saline; G3: 2.5 mg.kg^−1^ propofol; G4: 12.5 mg.kg^−1^ propofol; G5: 37.5 mg.kg^−1^ propofol; OGL: Outer granular layer; TC: Total cortex; PCC: Purkinje cell count.

**Table 2:** Findings obtained from histometric measurements made on spinal cord tissues on the 15th, 18th, and 21st day of incubation.

**15th day**	**GROUP**	**CE%**	**TO%**	**LU%**
	**G1**	**41.07 ± 1.10b**	**53.46 ± 0.97** a	**51.49 ± 0.84**
	**G2**	**42.80 ± 0.65b**	**51.31 ± 1.10** ab	**53.40 ± 0.60**
	**G3**	**41.81 ± 0.71b**	**54.66 ± 0.72** a	**50.89 ± 0.84**
	**G4**	**47.54 ± 1.18** a	**48.61 ± 0.86b**	**52.13 ± 2.21**
	**G5**	**43.58 ± 0.85b**	**51.38 ± 1.11** ab	**53.28 ± 0.75**
	***		***	**NS**
**18th day**	**G1**	**39.30 ± 0.61b**	**48.85 ± 1.12** a	**51.34 ± 0.91** ab
	**G2**	**36.65 ± 0.43c**	**45.89 ± 0.70** ab	**53.38 ± 0.68** a
	**G3**	**42.12 ± 0.72** a	**45.27 ± 0.84** ab	**50.93 ± 0.82** ab
	**G4**	**37.16 ± 0.54bc**	**43.61 ± 1.05b**	**52.94 ± 0.89** a
	**G5**	**36.47 ± 0.66c**	**44.34 ± 1.24b**	**48.87 ± 1.11b**
	***		**	**
**21st day**	**G1**	**37.31 ± 1.17**	**43.01 ± 1.33**	**49.29 ± 1.13b**
	**G2**	**36.09 ± 1.19**	**43.29 ± 1.03**	**52.94 ± 0.94** ab
	**G3**	**36.68 ± 0.66**	**44.82 ± 1.26**	**54.10 ± 1.06** a
	**G4**	**37.35 ± 1.05**	**45.41 ± 1.17**	**52.02 ± 0.86** ab
	**G5**	**37.94 ± 1.62**	**45.25 ± 1.24**	**54.78 ± 0.96** a
	**NS**		**NS**	**

NS: Not significant;

* p < 0.05;

** p < 0.01;

*** p < 0.001;

a, b Different letters in the same column are statistically significant;

G1: Control; G2: Saline; G3: 2.5 mg.kg^−1^ propofol; G4: 12.5 mg.kg^−1^ propofol; G5: 37.5 mg.kg^−1^ propofol; CE%: Substantia grisea in the cervical region in cross-sections ratio of total surface area; TO%: Ratio of substantia grisea in the thoracic region to the total surface area observed in the sections; LU%: Ratio of substantia grisea in the lumbar region to the total surface area observed in the sections.

**Table 3:** Values/findings obtained in transverse and longitudinal diameter measurements of canalis centralis in different segments of medulla spinalis on the 15th, 18th, and 21st day of incubation.

GROUP		CE		TO		LU	
		1 (transverse diameter)	2 (longitudinal diameter)	1 (transverse diameter)	2 (longitudinal diameter)	1 (transverse diameter)	2 (longitudinal diameter)
**15th day**	**G1**	17.99 ± 1.07^ab^	59.01 ± 4.26	26.98 ± 2.30^ab^	75.25 ± 5.23^a^	27.59 ± 1.61^b^	72.68 ± 3.46^b^
	**G2**	22.61 ± 0.80^a^	50.17 ± 3.42	30.63 ± 1.22^ab^	72.22 ± 4.85^a^	27.99 ± 2.03^ab^	**86.98 ± 4.24** a
	**G3**	21.53 ± 1.41^ab^	52.12 ± 1.34	22.72 ± 1.38^b^	76.09 ± 2.36^a^	34.11 ± 2.16^ab^	64.11 ± 2.01^b^
	**G4**	16.93 ± 1.69^b^	63.83 ± 5.68	32.02 ± 3.21^a^	**54.62 ± 4.86** b	**36.99 ± 2.77** a	73.19 ± 3.78^ab^
	**G5**	20.53 ± 1.87^ab^	62.82 ± 2.84	26.64 ± 2.18^ab^	69.37 ± 3.76^ab^	32.25 ± 2.67^ab^	63.97 ± 4.05^b^
		*	NS	*	**	*	***
**18th day**	**G1**	19.20 ± 0.78^b^	48.52 ± 2.13^ab^	25.24 ± 2.02^b^	63.13 ± 3.39	29.04 ± 2.91^b^	46.26 ± 4.32
	**G2**	24.08 ± 0.71^ab^	52.92 ± 2.69^a^	**34.02 ± 1.91** a	59.96 ± 2.17	**37.93 ± 2.39** a	54.25 ± 5.51
	**G3**	22.18 ± 1.32^ab^	47.32 ± 2.81^ab^	**32.64 ± 0.80** a	56.60 ± 1.56	23.33 ± 0.86^b^	41.33 ± 1.08
	**G4**	**28.38 ± 2.70** a	51.93 ± 4.00^ab^	29.64 ± 0.76^ab^	59.03 ± 1.82	28.85 ± 1.30^b^	50.35 ± 3.34
	**G5**	21.00 ± 1.65^b^	41.61 ± 2.31^b^	29.97 ± 1.19^ab^	57.22 ± 2.75	29.23 ± 2.39^b^	43.33 ± 2.67
		**	*	**	NS	***	NS
**21st day**	**G1**	24.04 ± 1.89b	43.92 ± 2.55^ab^	29.25 ± 1.54^ab^	43.06 ± 1.64	32.93 ± 1.19^ab^	32.81 ± 3.47^ab^
	**G2**	31.02 ± 1.23^ab^	46.31 ± 3.93^ab^	27.65 ± 1.87^ab^	45.56 ± 2.89	30.33 ± 2.70b	23.52 ± 3.23b
	**G3**	30.35 ± 1.12^ab^	33.67 ± 1.47b	32.41 ± 1.37^a^	38.37 ± 1.45	32.90 ± 2.35^ab^	35.06 ± 4.03^ab^
	**G4**	**34.21 ± 2.89** a	51.73 ± 5.57^a^	31.86 ± 1.61^a^	42.26 ± 2.24	39.79 ± 1.90^a^	37.80 ± 2.61^a^
	**G5**	**31.64 ± 1.23** a	47.20 ± 4.24^ab^	25.14 ± 0.98b	41.84 ± 2.23	30.84 ± 1.16b	30.17 ± 2.38^ab^
	**	*	**	NS	**	*	*

NS: Not significant;

* p < 0.05;

** p < 0.01;

*** p < 0.001;

a, b Different letters in the same column are statistically significant;

G1: Control; G2: Saline; G3: 2.5 mg.kg^−1^ propofol; G4: 12.5 mg.kg^−1^ propofol; G5: 37.5 mg.kg^−1^ propofol; CE: Cervical; TO: Thoracic; LU: Lumbar.
